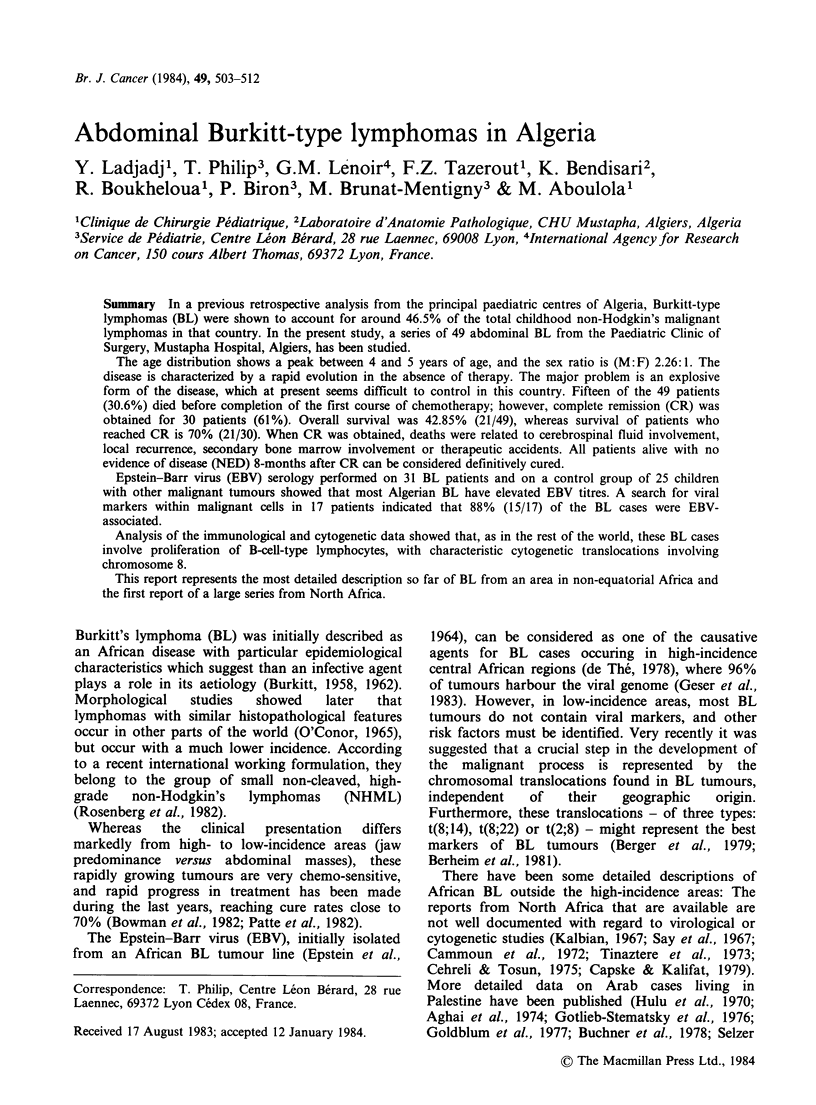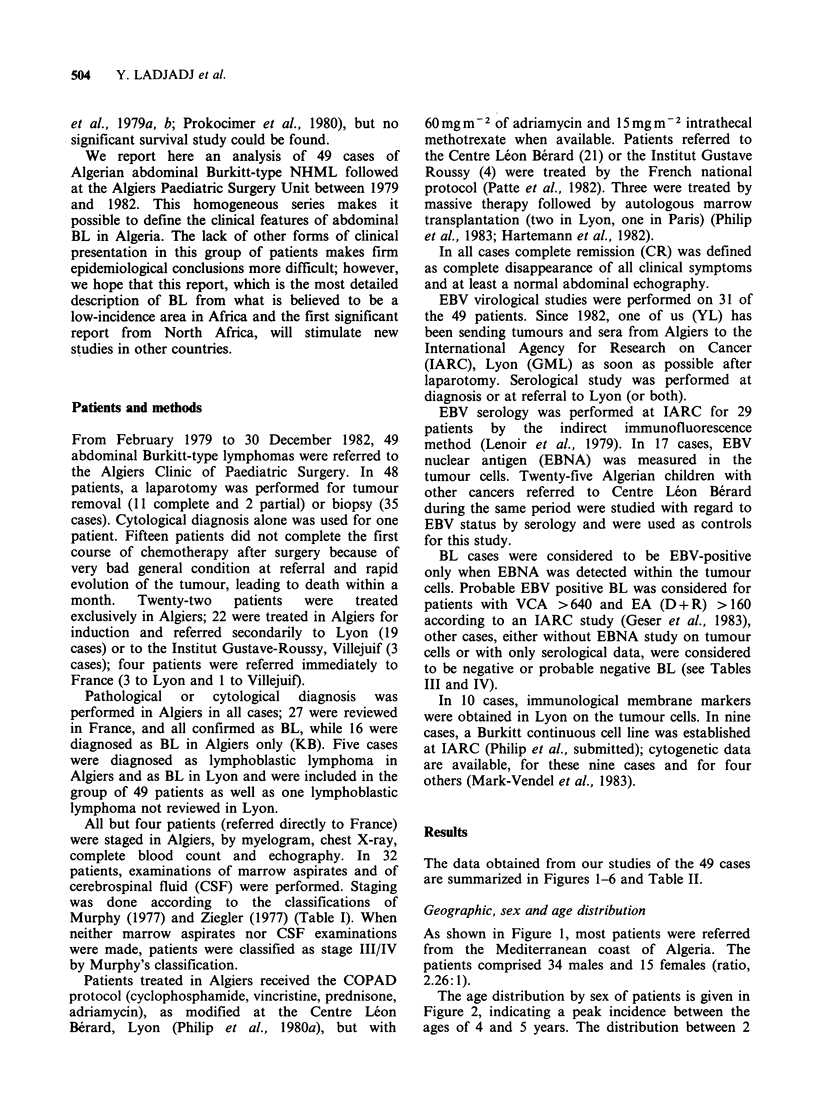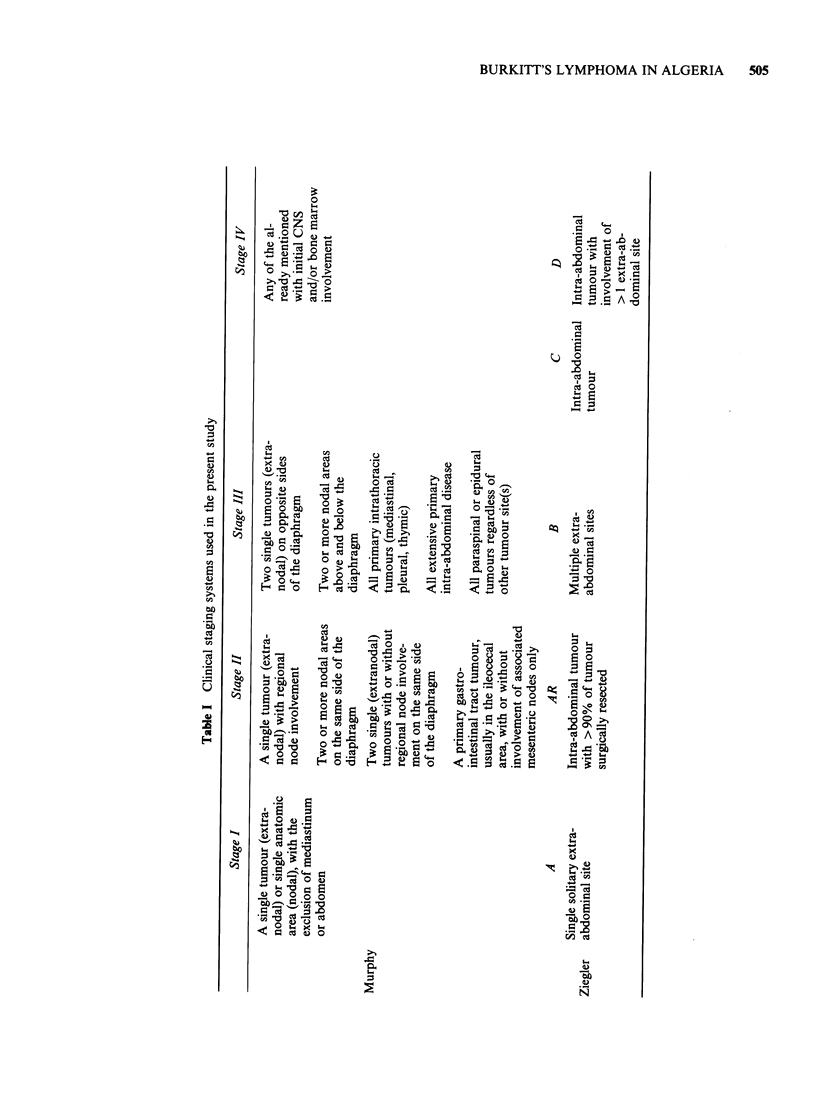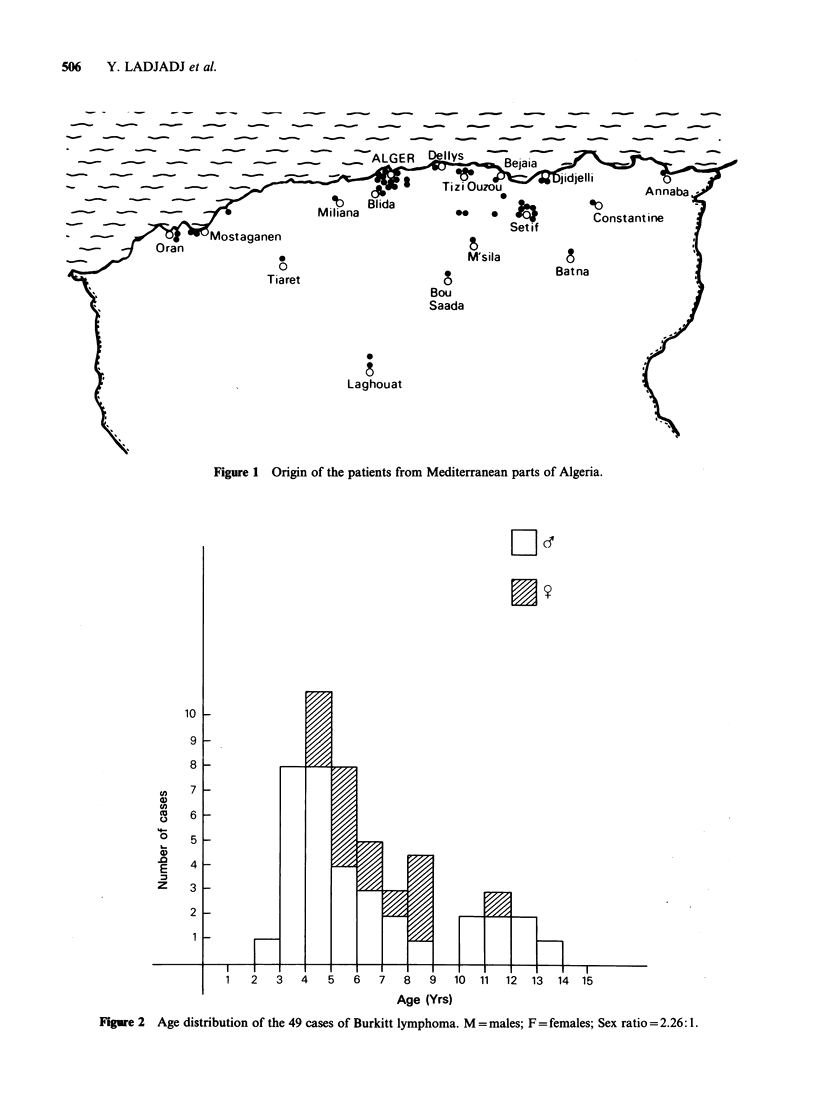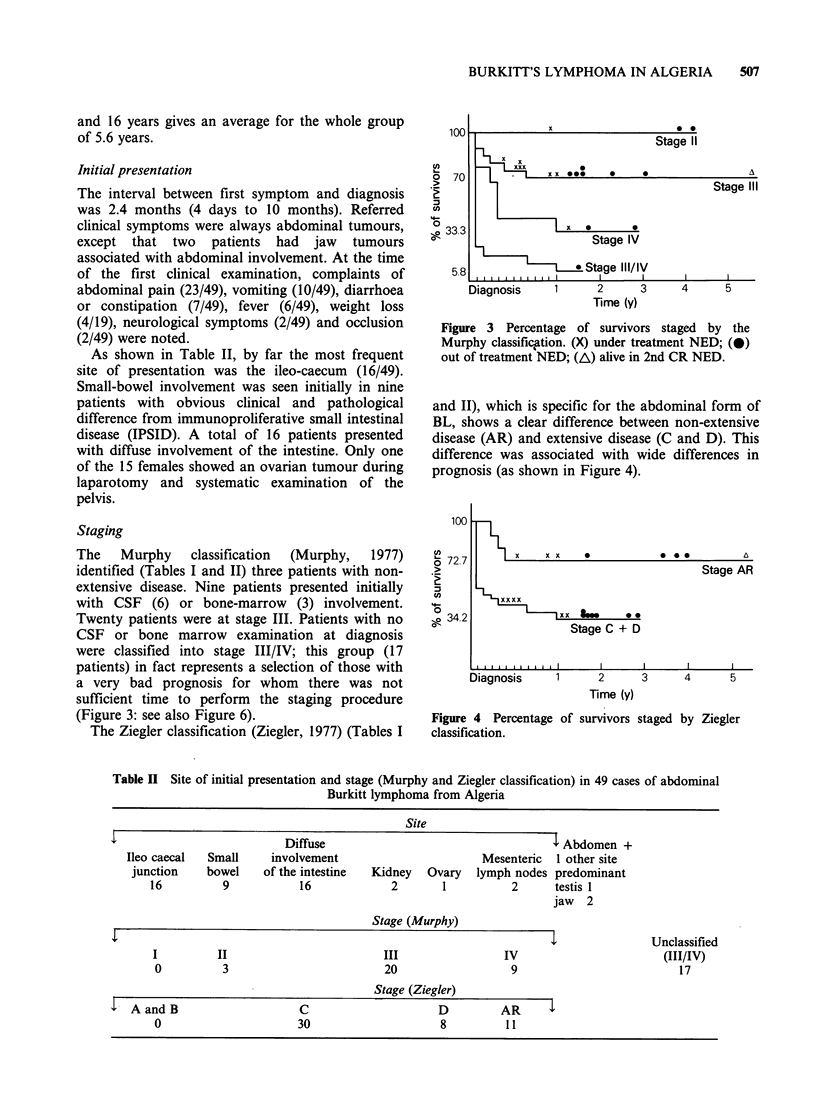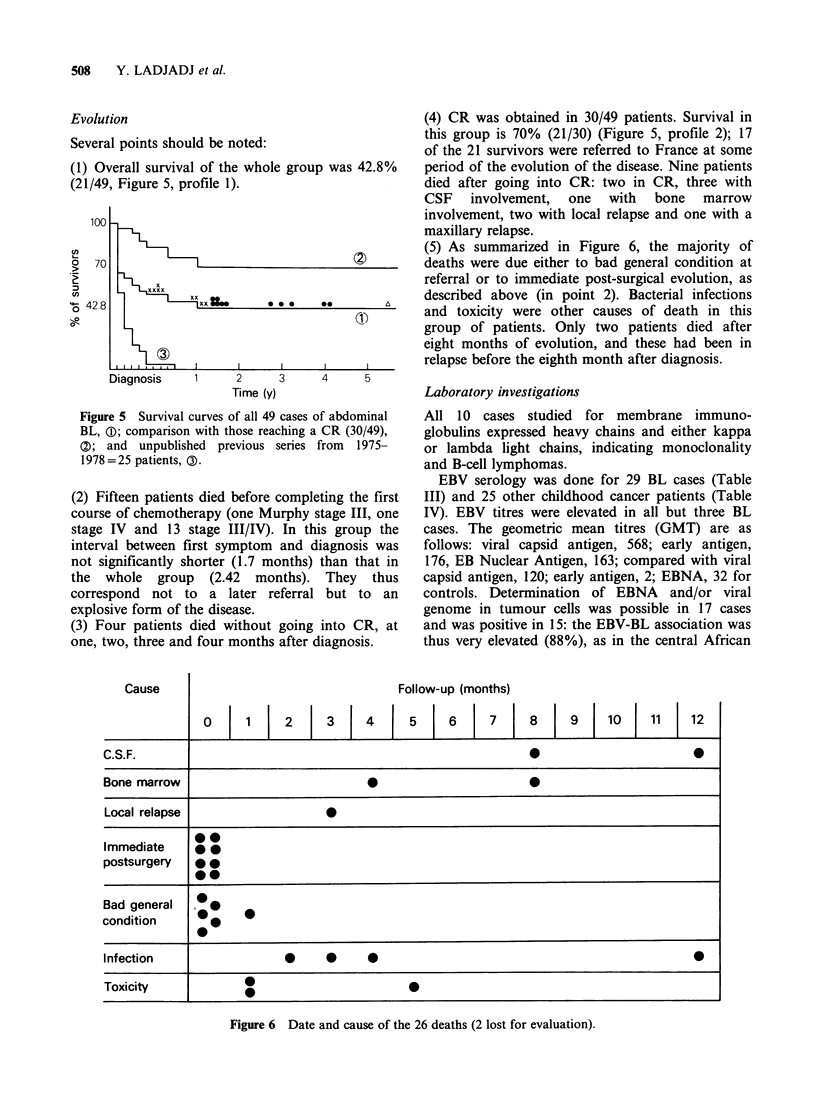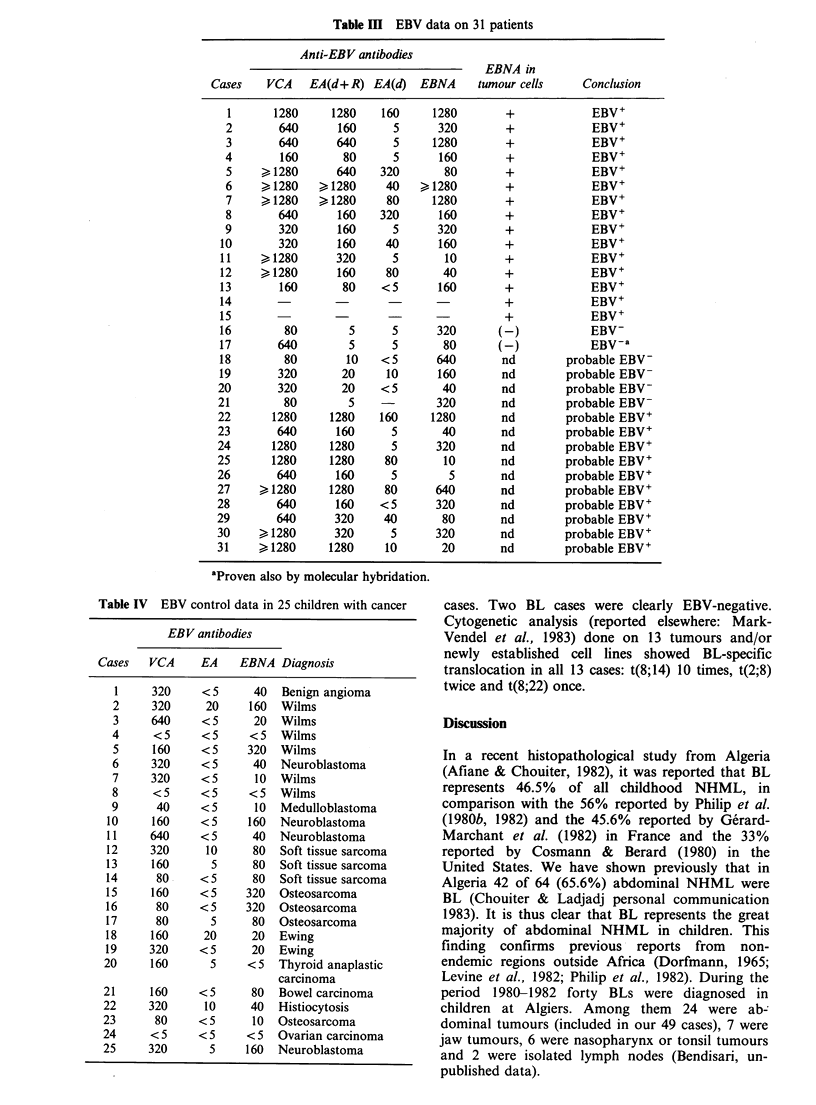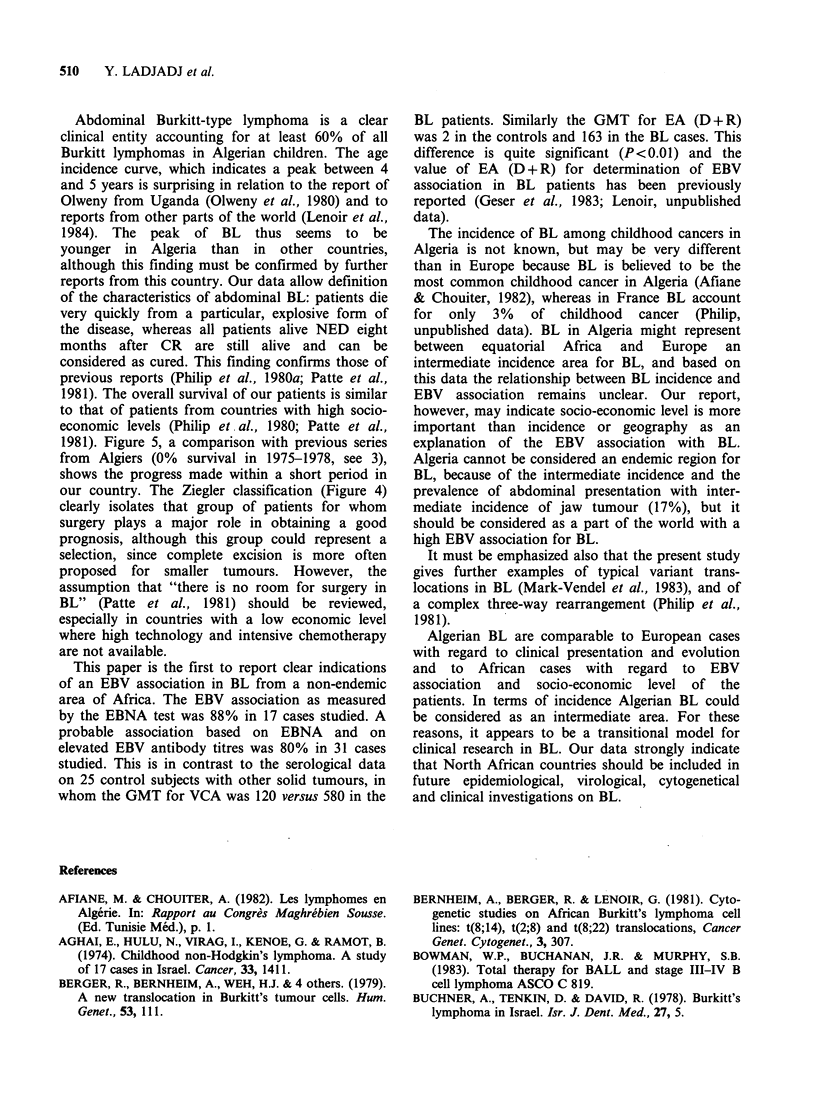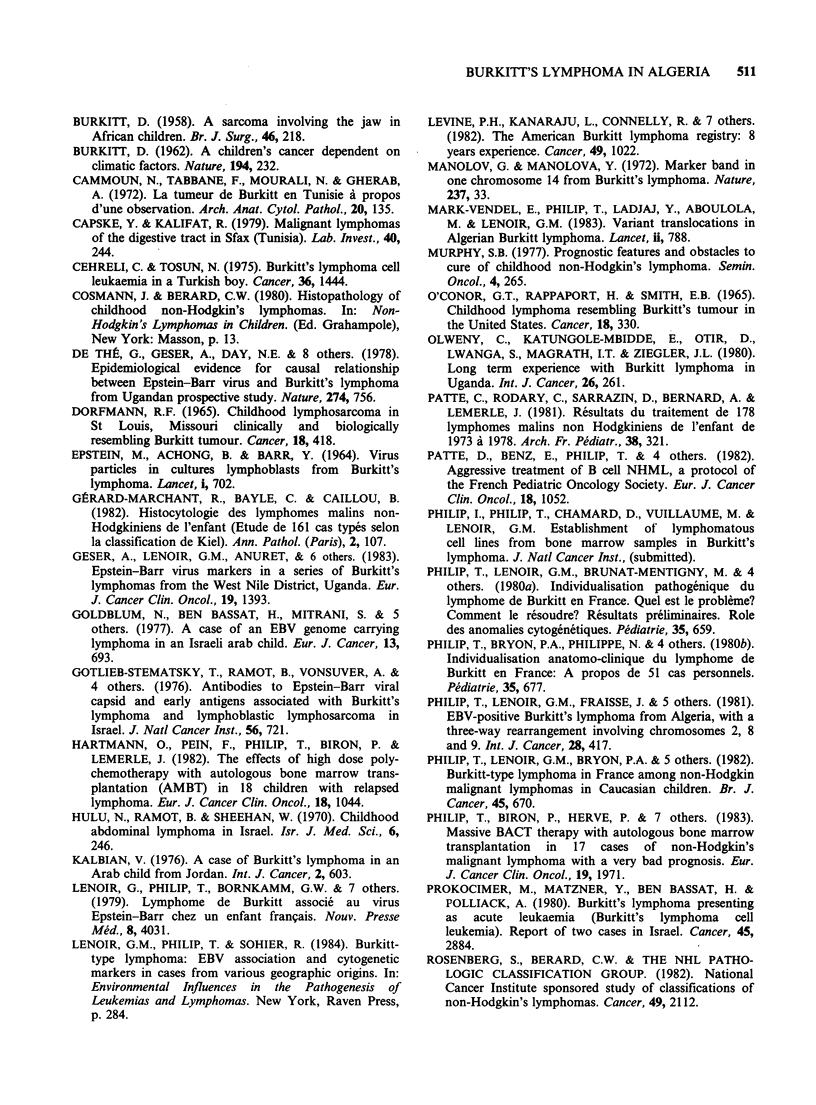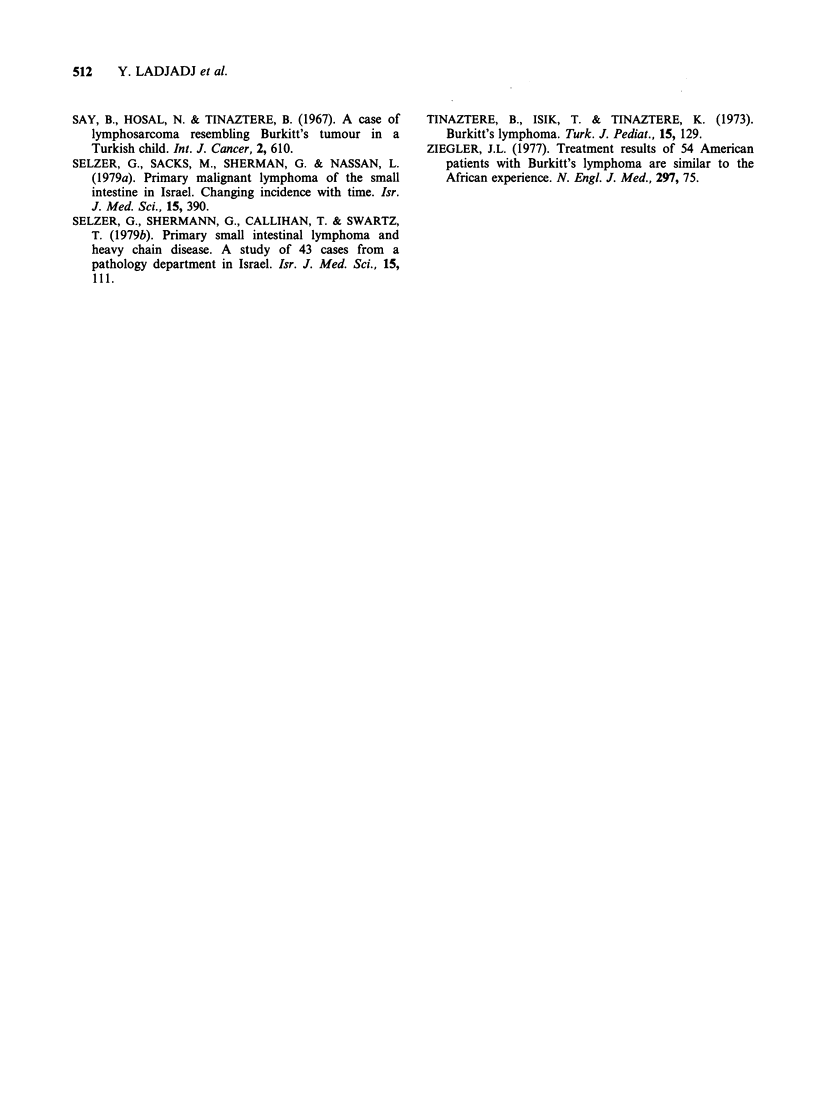# Abdominal Burkitt-type lymphomas in Algeria.

**DOI:** 10.1038/bjc.1984.78

**Published:** 1984-04

**Authors:** Y. Ladjadj, T. Philip, G. M. Lenoir, F. Z. Tazerout, K. Bendisari, R. Boukheloua, P. Biron, M. Brunat-Mentigny, M. Aboulola

## Abstract

In a previous retrospective analysis from the principal paediatric centres of Algeria, Burkitt-type lymphomas (BL) were shown to account for around 46.5% of the total childhood non-Hodgkin's malignant lymphomas in that country. In the present study, a series of 49 abdominal BL from the Paediatric Clinic of Surgery, Mustapha Hospital, Algiers, has been studied. The age distribution shows a peak between 4 and 5 years of age, and the sex ratio is (M:F) 2.26:1. The disease is characterized by a rapid evolution in the absence of therapy. The major problem is an explosive form of the disease, which at present seems difficult to control in this country. Fifteen of the 49 patients (30.6%) died before completion of the first course of chemotherapy; however, complete remission (CR) was obtained for 30 patients (61%). Overall survival was 42.85% (21/49), whereas survival of patients who reached CR is 70% (21/30). When CR was obtained, deaths were related to cerebrospinal fluid involvement, local recurrence, secondary bone marrow involvement or therapeutic accidents. All patients alive with no evidence of disease (NED) 8-months after CR can be considered definitively cured. Epstein-Barr virus (EBV) serology performed on 31 BL patients and on a control group of 25 children with other malignant tumours showed that most Algerian BL have elevated EBV titres. A search for viral markers within malignant cells in 17 patients indicated that 88% (15/17) of the BL cases were EBV-associated. Analysis of the immunological and cytogenetic data showed that, as in the rest of the world, these BL cases involve proliferation of B-cell-type lymphocytes, with characteristic cytogenetic translocations involving chromosome 8. This report represents the most detailed description so far of BL from an area in non-equatorial Africa and the first report of a large series from North Africa.


*Br. J. Cancer* (1984), **49**, 503–512

### Abdominal Burkitt-type lymphomas in Algeria

Y. Ladjadj[1], T. Philip[3], G.M. Lenoir[4], F.Z. Tazerout[1], K. Bendisari[2], R. Boukheloua[1], P. Biron[3], M. Brunat-Mentigny[3] & M. Aboulola[1]

[1]*Clinique de Chirurgie Pédiatrique,* [2]*Laboratoire d'Anatomie Pathologique, CHU Mustapha, Algiers, Algeria* [3]*Service de Pédiatrie, Centre Léon Bérard, 28 rue Laennec, 69008 Lyon,* [4]*International Agency for Research on Cancer, 150 cours Albert Thomas, 69372 Lyon, France.*

**Summary** In a previous retrospective analysis from the principal paediatric centres of Algeria, Burkitt-type lymphomas (BL) were shown to account for around 46.5% of the total childhood non-Hodgkin's malignant lymphomas in that country. In the present study, a series of 49 abdominal BL from the Paediatric Clinic of Surgery, Mustapha Hospital, Algiers, has been studied.

The age distribution shows a peak between 4 and 5 years of age, and the sex ratio is (M:F) 2.26:1. The disease is characterized by a rapid evolution in the absence of therapy. The major problem is an explosive form of the disease, which at present seems difficult to control in this country. Fifteen of the 49 patients (30.6%) died before completion of the first course of chemotherapy; however, complete remission (CR) was obtained for 30 patients (61%). Overall survival was 42.85% (21/49), whereas survival of patients who reached CR is 70% (21/30). When CR was obtained, deaths were related to cerebrospinal fluid involvement, local recurrence, secondary bone marrow involvement or therapeutic accidents. All patients alive with no evidence of disease (NED) 8-months after CR can be considered definitively cured.

Epstein–Barr virus (EBV) serology performed on 31 BL patients and on a control group of 25 children with other malignant tumours showed that most Algerian BL have elevated EBV titres. A search for viral markers within malignant cells in 17 patients indicated that 88% (15/17) of the BL cases were EBV-associated.

Analysis of the immunological and cytogenetic data showed that, as in the rest of the world, these BL cases involve proliferation of B-cell-type lymphocytes, with characteristic cytogenetic translocations involving chromosome 8.

This report represents the most detailed description so far of BL from an area in non-equatorial Africa and the first report of a large series from North Africa.

Burkitt's lymphoma (BL) was initially described as an African disease with particular epidemiological characteristics which suggest than an infective agent plays a role in its aetiology (Burkitt, 1958, 1962). Morphological studies showed later that lymphomas with similar histopathological features occur in other parts of the world (O'Conor, 1965), but occur with a much lower incidence. According to a recent international working formulation, they belong to the group of small non-cleaved, high-grade non-Hodgkin's lymphomas (NHML) (Rosenberg *et al.*, 1982).

Whereas the clinical presentation differs markedly from high- to low-incidence areas (jaw predominance *versus* abdominal masses), these rapidly growing tumours are very chemo-sensitive, and rapid progress in treatment has been made during the last years, reaching cure rates close to 70% (Bowman *et al.*, 1982; Patte *et al.*, 1982).

The Epstein–Barr virus (EBV), initially isolated from an African BL tumour line (Epstein *et al.*, 1964), can be considered as one of the causative agents for BL cases occuring in high-incidence central African regions (de Thé, 1978), where 96% of tumours harbour the viral genome (Geser *et al.*, 1983). However, in low-incidence areas, most BL tumours do not contain viral markers, and other risk factors must be identified. Very recently it was suggested that a crucial step in the development of the malignant process is represented by the chromosomal translocations found in BL tumours, independent of their geographic origin. Furthermore, these translocations – of three types: t(8;14), t(8;22) or t(2;8) – might represent the best markers of BL tumours (Berger *et al.*, 1979; Berheim *et al.*, 1981).

There have been some detailed descriptions of African BL outside the high-incidence areas: The reports from North Africa that are available are not well documented with regard to virological or cytogenetic studies (Kalbian, 1967; Say *et al.*, 1967; Cammoun *et al.*, 1972; Tinaztere *et al.*, 1973; Cehreli & Tosun, 1975; Capske & Kalifat, 1979). More detailed data on Arab cases living in Palestine have been published (Hulu *et al.*, 1970; Aghai *et al.*, 1974; Gotlieb-Stematsky *et al.*, 1976; Goldblum *et al.*, 1977; Buchner *et al.*, 1978; Selzer

Correspondence: T. Philip, Centre Léon Bérard, 28 rue Laennec, 69372 Lyon Cédex 08, France.

Received 17 August 1983; accepted 12 January 1984.

© The Macmillan Press Ltd., 1984

et al., 1979a, b; Prokocimer et al., 1980), but no significant survival study could be found.

We report here an analysis of 49 cases of Algerian abdominal Burkitt-type NHML followed at the Algiers Paediatric Surgery Unit between 1979 and 1982. This homogeneous series makes it possible to define the clinical features of abdominal BL in Algeria. The lack of other forms of clinical presentation in this group of patients makes firm epidemiological conclusions more difficult; however, we hope that this report, which is the most detailed description of BL from what is believed to be a low-incidence area in Africa and the first significant report from North Africa, will stimulate new studies in other countries.

### Patients and methods

From February 1979 to 30 December 1982, 49 abdominal Burkitt-type lymphomas were referred to the Algiers Clinic of Paediatric Surgery. In 48 patients, a laparotomy was performed for tumour removal (11 complete and 2 partial) or biopsy (35 cases). Cytological diagnosis alone was used for one patient. Fifteen patients did not complete the first course of chemotherapy after surgery because of very bad general condition at referral and rapid evolution of the tumour, leading to death within a month. Twenty-two patients were treated exclusively in Algiers; 22 were treated in Algiers for induction and referred secondarily to Lyon (19 cases) or to the Institut Gustave-Roussy, Villejuif (3 cases); four patients were referred immediately to France (3 to Lyon and 1 to Villejuif).

Pathological or cytological diagnosis was performed in Algiers in all cases; 27 were reviewed in France, and all confirmed as BL, while 16 were diagnosed as BL in Algiers only (KB). Five cases were diagnosed as lymphoblastic lymphoma in Algiers and as BL in Lyon and were included in the group of 49 patients as well as one lymphoblastic lymphoma not reviewed in Lyon.

All but four patients (referred directly to France) were staged in Algiers, by myelogram, chest X-ray, complete blood count and echography. In 32 patients, examinations of marrow aspirates and of cerebrospinal fluid (CSF) were performed. Staging was done according to the classifications of Murphy (1977) and Ziegler (1977) (Table I). When neither marrow aspirates nor CSF examinations were made, patients were classified as stage III/IV by Murphy's classification.

Patients treated in Algiers received the COPAD protocol (cyclophosphamide, vincristine, prednisone, adriamycin), as modified at the Centre Léon Bérard, Lyon (Philip et al., 1980a), but with $60\,mg\,m^{-2}$ of adriamycin and $15\,mg\,m^{-2}$ intrathecal methotrexate when available. Patients referred to the Centre Léon Bérard (21) or the Institut Gustave Roussy (4) were treated by the French national protocol (Patte et al., 1982). Three were treated by massive therapy followed by autologous marrow transplantation (two in Lyon, one in Paris) (Philip et al., 1983; Hartemann et al., 1982).

In all cases complete remission (CR) was defined as complete disappearance of all clinical symptoms and at least a normal abdominal echography.

EBV virological studies were performed on 31 of the 49 patients. Since 1982, one of us (YL) has been sending tumours and sera from Algiers to the International Agency for Research on Cancer (IARC), Lyon (GML) as soon as possible after laparotomy. Serological study was performed at diagnosis or at referral to Lyon (or both).

EBV serology was performed at IARC for 29 patients by the indirect immunofluorescence method (Lenoir et al., 1979). In 17 cases, EBV nuclear antigen (EBNA) was measured in the tumour cells. Twenty-five Algerian children with other cancers referred to Centre Léon Bérard during the same period were studied with regard to EBV status by serology and were used as controls for this study.

BL cases were considered to be EBV-positive only when EBNA was detected within the tumour cells. Probable EBV positive BL was considered for patients with VCA $>640$ and EA (D+R) $>160$ according to an IARC study (Geser et al., 1983), other cases, either without EBNA study on tumour cells or with only serological data, were considered to be negative or probable negative BL (see Tables III and IV).

In 10 cases, immunological membrane markers were obtained in Lyon on the tumour cells. In nine cases, a Burkitt continuous cell line was established at IARC (Philip et al., submitted); cytogenetic data are available, for these nine cases and for four others (Mark-Vendel et al., 1983).

### Results

The data obtained from our studies of the 49 cases are summarized in Figures 1–6 and Table II.

### Geographic, sex and age distribution

As shown in Figure 1, most patients were referred from the Mediterranean coast of Algeria. The patients comprised 34 males and 15 females (ratio, 2.26:1).

The age distribution by sex of patients is given in Figure 2, indicating a peak incidence between the ages of 4 and 5 years. The distribution between 2

**Table I** Clinical staging systems used in the present study

| | *Stage I* | *Stage II* | *Stage III* | | *Stage IV* |
|---|---|---|---|---|---|
| Murphy | A single tumour (extra-nodal) or single anatomic area (nodal), with the exclusion of mediastinum or abdomen | A single tumour (extra-nodal) with regional node involvement<br><br>Two or more nodal areas on the same side of the diaphragm<br><br>Two single (extranodal) tumours with or without regional node involvement on the same side of the diaphragm<br><br>A primary gastro-intestinal tract tumour, usually in the ileocecal area, with or without involvement of associated mesenteric nodes only | Two single tumours (extra-nodal) on opposite sides of the diaphragm<br><br>Two or more nodal areas above and below the diaphragm<br><br>All primary intrathoracic tumours (mediastinal, pleural, thymic)<br><br>All extensive primary intra-abdominal disease<br><br>All paraspinal or epidural tumours regardless of other tumour site(s) | | Any of the already mentioned with initial CNS and/or bone marrow involvement |
| | *A* | *AR* | *B* | *C* | *D* |
| Ziegler | Single solitary extra-abdominal site | Intra-abdominal tumour with >90% of tumour surgically resected | Multiple extra-abdominal sites | Intra-abdominal tumour | Intra-abdominal tumour with involvement of >1 extra-abdominal site |

**Figure 1** Origin of the patients from Mediterranean parts of Algeria.

**Figure 2** Age distribution of the 49 cases of Burkitt lymphoma. M = males; F = females; Sex ratio = 2.26:1.

and 16 years gives an average for the whole group of 5.6 years.

*Initial presentation*

The interval between first symptom and diagnosis was 2.4 months (4 days to 10 months). Referred clinical symptoms were always abdominal tumours, except that two patients had jaw tumours associated with abdominal involvement. At the time of the first clinical examination, complaints of abdominal pain (23/49), vomiting (10/49), diarrhoea or constipation (7/49), fever (6/49), weight loss (4/19), neurological symptoms (2/49) and occlusion (2/49) were noted.

As shown in Table II, by far the most frequent site of presentation was the ileo-caecum (16/49). Small-bowel involvement was seen initially in nine patients with obvious clinical and pathological difference from immunoproliferative small intestinal disease (IPSID). A total of 16 patients presented with diffuse involvement of the intestine. Only one of the 15 females showed an ovarian tumour during laparotomy and systematic examination of the pelvis.

*Staging*

The Murphy classification (Murphy, 1977) identified (Tables I and II) three patients with non-extensive disease. Nine patients presented initially with CSF (6) or bone-marrow (3) involvement. Twenty patients were at stage III. Patients with no CSF or bone marrow examination at diagnosis were classified into stage III/IV; this group (17 patients) in fact represents a selection of those with a very bad prognosis for whom there was not sufficient time to perform the staging procedure (Figure 3: see also Figure 6).

The Ziegler classification (Ziegler, 1977) (Tables I and II), which is specific for the abdominal form of BL, shows a clear difference between non-extensive disease (AR) and extensive disease (C and D). This difference was associated with wide differences in prognosis (as shown in Figure 4).

**Figure 3** Percentage of survivors staged by the Murphy classification. (X) under treatment NED; (●) out of treatment NED; (△) alive in 2nd CR NED.

**Figure 4** Percentage of survivors staged by Ziegler classification.

**Table II** Site of initial presentation and stage (Murphy and Ziegler classification) in 49 cases of abdominal Burkitt lymphoma from Algeria

*Site*

| Ileo caecal junction | Small bowel | Diffuse involvement of the intestine | Kidney | Ovary | Mesenteric lymph nodes | Abdomen + 1 other site predominant |
|---|---|---|---|---|---|---|
| 16 | 9 | 16 | 2 | 1 | 2 | testis 1<br>jaw 2 |

*Stage (Murphy)*

| I | II | III | IV | Unclassified (III/IV) |
|---|---|---|---|---|
| 0 | 3 | 20 | 9 | 17 |

*Stage (Ziegler)*

| A and B | C | D | AR |
|---|---|---|---|
| 0 | 30 | 8 | 11 |

*Evolution*

Several points should be noted:

(1) Overall survival of the whole group was 42.8% (21/49, Figure 5, profile 1).

**Figure 5** Survival curves of all 49 cases of abdominal BL, ①; comparison with those reaching a CR (30/49), ②; and unpublished previous series from 1975–1978 = 25 patients, ③.

(2) Fifteen patients died before completing the first course of chemotherapy (one Murphy stage III, one stage IV and 13 stage III/IV). In this group the interval between first symptom and diagnosis was not significantly shorter (1.7 months) than that in the whole group (2.42 months). They thus correspond not to a later referral but to an explosive form of the disease.

(3) Four patients died without going into CR, at one, two, three and four months after diagnosis.

(4) CR was obtained in 30/49 patients. Survival in this group is 70% (21/30) (Figure 5, profile 2); 17 of the 21 survivors were referred to France at some period of the evolution of the disease. Nine patients died after going into CR: two in CR, three with CSF involvement, one with bone marrow involvement, two with local relapse and one with a maxillary relapse.

(5) As summarized in Figure 6, the majority of deaths were due either to bad general condition at referral or to immediate post-surgical evolution, as described above (in point 2). Bacterial infections and toxicity were other causes of death in this group of patients. Only two patients died after eight months of evolution, and these had been in relapse before the eighth month after diagnosis.

*Laboratory investigations*

All 10 cases studied for membrane immunoglobulins expressed heavy chains and either kappa or lambda light chains, indicating monoclonality and B-cell lymphomas.

EBV serology was done for 29 BL cases (Table III) and 25 other childhood cancer patients (Table IV). EBV titres were elevated in all but three BL cases. The geometric mean titres (GMT) are as follows: viral capsid antigen, 568; early antigen, 176, EB Nuclear Antigen, 163; compared with viral capsid antigen, 120; early antigen, 2; EBNA, 32 for controls. Determination of EBNA and/or viral genome in tumour cells was possible in 17 cases and was positive in 15: the EBV-BL association was thus very elevated (88%), as in the central African

**Figure 6** Date and cause of the 26 deaths (2 lost for evaluation).

**Table III** EBV data on 31 patients

| Cases | *Anti-EBV antibodies* VCA | EA(d+R) | EA(d) | EBNA | *EBNA in tumour cells* | *Conclusion* |
|---|---|---|---|---|---|---|
| 1 | 1280 | 1280 | 160 | 1280 | + | $EBV^+$ |
| 2 | 640 | 160 | 5 | 320 | + | $EBV^+$ |
| 3 | 640 | 640 | 5 | 1280 | + | $EBV^+$ |
| 4 | 160 | 80 | 5 | 160 | + | $EBV^+$ |
| 5 | ⩾1280 | 640 | 320 | 80 | + | $EBV^+$ |
| 6 | ⩾1280 | ⩾1280 | 40 | ⩾1280 | + | $EBV^+$ |
| 7 | ⩾1280 | ⩾1280 | 80 | 1280 | + | $EBV^+$ |
| 8 | 640 | 160 | 320 | 160 | + | $EBV^+$ |
| 9 | 320 | 160 | 5 | 320 | + | $EBV^+$ |
| 10 | 320 | 160 | 40 | 160 | + | $EBV^+$ |
| 11 | ⩾1280 | 320 | 5 | 10 | + | $EBV^+$ |
| 12 | ⩾1280 | 160 | 80 | 40 | + | $EBV^+$ |
| 13 | 160 | 80 | <5 | 160 | + | $EBV^+$ |
| 14 | — | — | — | — | + | $EBV^+$ |
| 15 | — | — | — | — | + | $EBV^+$ |
| 16 | 80 | 5 | 5 | 320 | (−) | $EBV^-$ |
| 17 | 640 | 5 | 5 | 80 | (−) | $EBV^{-a}$ |
| 18 | 80 | 10 | <5 | 640 | nd | probable $EBV^-$ |
| 19 | 320 | 20 | 10 | 160 | nd | probable $EBV^-$ |
| 20 | 320 | 20 | <5 | 40 | nd | probable $EBV^-$ |
| 21 | 80 | 5 | — | 320 | nd | probable $EBV^-$ |
| 22 | 1280 | 1280 | 160 | 1280 | nd | probable $EBV^+$ |
| 23 | 640 | 160 | 5 | 40 | nd | probable $EBV^+$ |
| 24 | 1280 | 1280 | 5 | 320 | nd | probable $EBV^+$ |
| 25 | 1280 | 1280 | 80 | 10 | nd | probable $EBV^+$ |
| 26 | 640 | 160 | 5 | 5 | nd | probable $EBV^+$ |
| 27 | ⩾1280 | 1280 | 80 | 640 | nd | probable $EBV^+$ |
| 28 | 640 | 160 | <5 | 320 | nd | probable $EBV^+$ |
| 29 | 640 | 320 | 40 | 80 | nd | probable $EBV^+$ |
| 30 | ⩾1280 | 320 | 5 | 320 | nd | probable $EBV^+$ |
| 31 | ⩾1280 | 1280 | 10 | 20 | nd | probable $EBV^+$ |

[a]Proven also by molecular hybridation.

**Table IV** EBV control data in 25 children with cancer

| Cases | *EBV antibodies* VCA | EA | EBNA | Diagnosis |
|---|---|---|---|---|
| 1 | 320 | <5 | 40 | Benign angioma |
| 2 | 320 | 20 | 160 | Wilms |
| 3 | 640 | <5 | 20 | Wilms |
| 4 | <5 | <5 | <5 | Wilms |
| 5 | 160 | <5 | 320 | Wilms |
| 6 | 320 | <5 | 40 | Neuroblastoma |
| 7 | 320 | <5 | 10 | Wilms |
| 8 | <5 | <5 | <5 | Wilms |
| 9 | 40 | <5 | 10 | Medulloblastoma |
| 10 | 160 | <5 | 160 | Neuroblastoma |
| 11 | 640 | <5 | 40 | Neuroblastoma |
| 12 | 320 | 10 | 80 | Soft tissue sarcoma |
| 13 | 160 | 5 | 80 | Soft tissue sarcoma |
| 14 | 80 | <5 | 80 | Soft tissue sarcoma |
| 15 | 160 | <5 | 320 | Osteosarcoma |
| 16 | 80 | <5 | 320 | Osteosarcoma |
| 17 | 80 | 5 | 80 | Osteosarcoma |
| 18 | 160 | 20 | 20 | Ewing |
| 19 | 320 | <5 | 20 | Ewing |
| 20 | 160 | 5 | <5 | Thyroid anaplastic carcinoma |
| 21 | 160 | <5 | 80 | Bowel carcinoma |
| 22 | 320 | 10 | 40 | Histiocytosis |
| 23 | 80 | <5 | 10 | Osteosarcoma |
| 24 | <5 | <5 | <5 | Ovarian carcinoma |
| 25 | 320 | 5 | 160 | Neuroblastoma |

cases. Two BL cases were clearly EBV-negative. Cytogenetic analysis (reported elsewhere: Mark-Vendel *et al.*, 1983) done on 13 tumours and/or newly established cell lines showed BL-specific translocation in all 13 cases: t(8;14) 10 times, t(2;8) twice and t(8;22) once.

### Discussion

In a recent histopathological study from Algeria (Afiane & Chouiter, 1982), it was reported that BL represents 46.5% of all childhood NHML, in comparison with the 56% reported by Philip *et al.* (1980*b*, 1982) and the 45.6% reported by Gérard-Marchant *et al.* (1982) in France and the 33% reported by Cosmann & Berard (1980) in the United States. We have shown previously that in Algeria 42 of 64 (65.6%) abdominal NHML were BL (Chouiter & Ladjadj personal communication 1983). It is thus clear that BL represents the great majority of abdominal NHML in children. This finding confirms previous reports from non-endemic regions outside Africa (Dorfmann, 1965; Levine *et al.*, 1982; Philip *et al.*, 1982). During the period 1980–1982 forty BLs were diagnosed in children at Algiers. Among them 24 were abdominal tumours (included in our 49 cases), 7 were jaw tumours, 6 were nasopharynx or tonsil tumours and 2 were isolated lymph nodes (Bendisari, unpublished data).

Abdominal Burkitt-type lymphoma is a clear clinical entity accounting for at least 60% of all Burkitt lymphomas in Algerian children. The age incidence curve, which indicates a peak between 4 and 5 years is surprising in relation to the report of Olweny from Uganda (Olweny *et al.*, 1980) and to reports from other parts of the world (Lenoir *et al.*, 1984). The peak of BL thus seems to be younger in Algeria than in other countries, although this finding must be confirmed by further reports from this country. Our data allow definition of the characteristics of abdominal BL: patients die very quickly from a particular, explosive form of the disease, whereas all patients alive NED eight months after CR are still alive and can be considered as cured. This finding confirms those of previous reports (Philip *et al.*, 1980*a*; Patte *et al.*, 1981). The overall survival of our patients is similar to that of patients from countries with high socio-economic levels (Philip *et al.*, 1980; Patte *et al.*, 1981). Figure 5, a comparison with previous series from Algiers (0% survival in 1975–1978, see 3), shows the progress made within a short period in our country. The Ziegler classification (Figure 4) clearly isolates that group of patients for whom surgery plays a major role in obtaining a good prognosis, although this group could represent a selection, since complete excision is more often proposed for smaller tumours. However, the assumption that "there is no room for surgery in BL" (Patte *et al.*, 1981) should be reviewed, especially in countries with a low economic level where high technology and intensive chemotherapy are not available.

This paper is the first to report clear indications of an EBV association in BL from a non-endemic area of Africa. The EBV association as measured by the EBNA test was 88% in 17 cases studied. A probable association based on EBNA and on elevated EBV antibody titres was 80% in 31 cases studied. This is in contrast to the serological data on 25 control subjects with other solid tumours, in whom the GMT for VCA was 120 *versus* 580 in the BL patients. Similarly the GMT for EA (D+R) was 2 in the controls and 163 in the BL cases. This difference is quite significant ($P<0.01$) and the value of EA (D+R) for determination of EBV association in BL patients has been previously reported (Geser *et al.*, 1983; Lenoir, unpublished data).

The incidence of BL among childhood cancers in Algeria is not known, but may be very different than in Europe because BL is believed to be the most common childhood cancer in Algeria (Afiane & Chouiter, 1982), whereas in France BL account for only 3% of childhood cancer (Philip, unpublished data). BL in Algeria might represent between equatorial Africa and Europe an intermediate incidence area for BL, and based on this data the relationship between BL incidence and EBV association remains unclear. Our report, however, may indicate socio-economic level is more important than incidence or geography as an explanation of the EBV association with BL. Algeria cannot be considered an endemic region for BL, because of the intermediate incidence and the prevalence of abdominal presentation with intermediate incidence of jaw tumour (17%), but it should be considered as a part of the world with a high EBV association for BL.

It must be emphasized also that the present study gives further examples of typical variant translocations in BL (Mark-Vendel *et al.*, 1983), and of a complex three-way rearrangement (Philip *et al.*, 1981).

Algerian BL are comparable to European cases with regard to clinical presentation and evolution and to African cases with regard to EBV association and socio-economic level of the patients. In terms of incidence Algerian BL could be considered as an intermediate area. For these reasons, it appears to be a transitional model for clinical research in BL. Our data strongly indicate that North African countries should be included in future epidemiological, virological, cytogenetical and clinical investigations on BL.

### References

AFIANE, M. & CHOUITER, A. (1982). Les lymphomes en Algérie. In: *Rapport au Congrès Maghrébien Sousse.* (Ed. Tunisie Méd.), p. 1.

AGHAI, E., HULU, N., VIRAG, I., KENOE, G. & RAMOT, B. (1974). Childhood non-Hodgkin's lymphoma. A study of 17 cases in Israel. *Cancer,* **33,** 1411.

BERGER, R., BERNHEIM, A., WEH, H.J. & 4 others. (1979). A new translocation in Burkitt's tumour cells. *Hum. Genet.,* **53,** 111.

BERNHEIM, A., BERGER, R. & LENOIR, G. (1981). Cytogenetic studies on African Burkitt's lymphoma cell lines: t(8;14), t(2;8) and t(8;22) translocations, *Cancer Genet. Cytogenet.,* **3,** 307.

BOWMAN, W.P., BUCHANAN, J.R. & MURPHY, S.B. (1983). Total therapy for BALL and stage III–IV B cell lymphoma ASCO C 819.

BUCHNER, A., TENKIN, D. & DAVID, R. (1978). Burkitt's lymphoma in Israel. *Isr. J. Dent. Med.,* **27,** 5.

BURKITT, D. (1958). A sarcoma involving the jaw in African children. *Br. J. Surg.*, **46**, 218.

BURKITT, D. (1962). A children's cancer dependent on climatic factors. *Nature*, **194**, 232.

CAMMOUN, N., TABBANE, F., MOURALI, N. & GHERAB, A. (1972). La tumeur de Burkitt en Tunisie à propos d'une observation. *Arch. Anat. Cytol. Pathol.*, **20**, 135.

CAPSKE, Y. & KALIFAT, R. (1979). Malignant lymphomas of the digestive tract in Sfax (Tunisia). *Lab. Invest.*, **40**, 244.

CEHRELI, C. & TOSUN, N. (1975). Burkitt's lymphoma cell leukaemia in a Turkish boy. *Cancer*, **36**, 1444.

COSMANN, J. & BERARD, C.W. (1980). Histopathology of childhood non-Hodgkin's lymphomas. In: *Non-Hodgkin's Lymphomas in Children*. (Ed. Grahampole), New York: Masson, p. 13.

DE THÉ, G., GESER, A., DAY, N.E. & 8 others. (1978). Epidemiological evidence for causal relationship between Epstein–Barr virus and Burkitt's lymphoma from Ugandan prospective study. *Nature*, **274**, 756.

DORFMANN, R.F. (1965). Childhood lymphosarcoma in St Louis, Missouri clinically and biologically resembling Burkitt tumour. *Cancer*, **18**, 418.

EPSTEIN, M., ACHONG, B. & BARR, Y. (1964). Virus particles in cultures lymphoblasts from Burkitt's lymphoma. *Lancet*, **i**, 702.

GÉRARD-MARCHANT, R., BAYLE, C. & CAILLOU, B. (1982). Histocytologie des lymphomes malins non-Hodgkiniens de l'enfant (Etude de 161 cas typés selon la classification de Kiel). *Ann. Pathol. (Paris)*, **2**, 107.

GESER, A., LENOIR, G.M., ANURET, & 6 others. (1983). Epstein–Barr virus markers in a series of Burkitt's lymphomas from the West Nile District, Uganda. *Eur. J. Cancer Clin. Oncol.*, **19**, 1393.

GOLDBLUM, N., BEN BASSAT, H., MITRANI, S. & 5 others. (1977). A case of an EBV genome carrying lymphoma in an Israeli arab child. *Eur. J. Cancer*, **13**, 693.

GOTLIEB-STEMATSKY, T., RAMOT, B., VONSUVER, A. & 4 others. (1976). Antibodies to Epstein–Barr viral capsid and early antigens associated with Burkitt's lymphoma and lymphoblastic lymphosarcoma in Israel. *J. Natl Cancer Inst.*, **56**, 721.

HARTMANN, O., PEIN, F., PHILIP, T., BIRON, P. & LEMERLE, J. (1982). The effects of high dose polychemotherapy with autologous bone marrow transplantation (AMBT) in 18 children with relapsed lymphoma. *Eur. J. Cancer Clin. Oncol.*, **18**, 1044.

HULU, N., RAMOT, B. & SHEEHAN, W. (1970). Childhood abdominal lymphoma in Israel. *Isr. J. Med. Sci.*, **6**, 246.

KALBIAN, V. (1976). A case of Burkitt's lymphoma in an Arab child from Jordan. *Int. J. Cancer*, **2**, 603.

LENOIR, G., PHILIP, T., BORNKAMM, G.W. & 7 others. (1979). Lymphome de Burkitt associé au virus Epstein–Barr chez un enfant français. *Nouv. Presse Méd.*, **8**, 4031.

LENOIR, G.M., PHILIP, T. & SOHIER, R. (1984). Burkitt-type lymphoma: EBV association and cytogenetic markers in cases from various geographic origins. In: *Environmental Influences in the Pathogenesis of Leukemias and Lymphomas*. New York, Raven Press, p. 284.

LEVINE, P.H., KANARAJU, L., CONNELLY, R. & 7 others. (1982). The American Burkitt lymphoma registry: 8 years experience. *Cancer*, **49**, 1022.

MANOLOV, G. & MANOLOVA, Y. (1972). Marker band in one chromosome 14 from Burkitt's lymphoma. *Nature*, **237**, 33.

MARK-VENDEL, E., PHILIP, T., LADJAJ, Y., ABOULOLA, M. & LENOIR, G.M. (1983). Variant translocations in Algerian Burkitt lymphoma. *Lancet*, **ii**, 788.

MURPHY, S.B. (1977). Prognostic features and obstacles to cure of childhood non-Hodgkin's lymphoma. *Semin. Oncol.*, **4**, 265.

O'CONOR, G.T., RAPPAPORT, H. & SMITH, E.B. (1965). Childhood lymphoma resembling Burkitt's tumour in the United States. *Cancer*, **18**, 330.

OLWENY, C., KATUNGOLE-MBIDDE, E., OTIR, D., LWANGA, S., MAGRATH, I.T. & ZIEGLER, J.L. (1980). Long term experience with Burkitt lymphoma in Uganda. *Int. J. Cancer*, **26**, 261.

PATTE, C., RODARY, C., SARRAZIN, D., BERNARD, A. & LEMERLE, J. (1981). Résultats du traitement de 178 lymphomes malins non Hodgkiniens de l'enfant de 1973 à 1978. *Arch. Fr. Pédiatr.*, **38**, 321.

PATTE, D., BENZ, E., PHILIP, T. & 4 others. (1982). Aggressive treatment of B cell NHML, a protocol of the French Pediatric Oncology Society. *Eur. J. Cancer Clin. Oncol.*, **18**, 1052.

PHILIP, I., PHILIP, T., CHAMARD, D., VUILLAUME, M. & LENOIR, G.M. Establishment of lymphomatous cell lines from bone marrow samples in Burkitt's lymphoma. *J. Natl Cancer Inst.*, (submitted).

PHILIP, T., LENOIR, G.M., BRUNAT-MENTIGNY, M. & 4 others. (1980*a*). Individualisation pathogénique du lymphome de Burkitt en France. Quel est le problème? Comment le résoudre? Résultats préliminaires. Role des anomalies cytogénétiques. *Pédiatrie*, **35**, 659.

PHILIP, T., BRYON, P.A., PHILIPPE, N. & 4 others. (1980*b*). Individualisation anatomo-clinique du lymphome de Burkitt en France: A propos de 51 cas personnels. *Pédiatrie*, **35**, 677.

PHILIP, T., LENOIR, G.M., FRAISSE, J. & 5 others. (1981). EBV-positive Burkitt's lymphoma from Algeria, with a three-way rearrangement involving chromosomes 2, 8 and 9. *Int. J. Cancer*, **28**, 417.

PHILIP, T., LENOIR, G.M., BRYON, P.A. & 5 others. (1982). Burkitt-type lymphoma in France among non-Hodgkin malignant lymphomas in Caucasian children. *Br. J. Cancer*, **45**, 670.

PHILIP, T., BIRON, P., HERVE, P. & 7 others. (1983). Massive BACT therapy with autologous bone marrow transplantation in 17 cases of non-Hodgkin's malignant lymphoma with a very bad prognosis. *Eur. J. Cancer Clin. Oncol.*, **19**, 1971.

PROKOCIMER, M., MATZNER, Y., BEN BASSAT, H. & POLLIACK, A. (1980). Burkitt's lymphoma presenting as acute leukaemia (Burkitt's lymphoma cell leukemia). Report of two cases in Israel. *Cancer*, **45**, 2884.

ROSENBERG, S., BERARD, C.W. & THE NHL PATHOLOGIC CLASSIFICATION GROUP. (1982). National Cancer Institute sponsored study of classifications of non-Hodgkin's lymphomas. *Cancer*, **49**, 2112.

SAY, B., HOSAL, N. & TINAZTERE, B. (1967). A case of lymphosarcoma resembling Burkitt's tumour in a Turkish child. *Int. J. Cancer,* **2,** 610.

SELZER, G., SACKS, M., SHERMAN, G. & NASSAN, L. (1979*a*). Primary malignant lymphoma of the small intestine in Israel. Changing incidence with time. *Isr. J. Med. Sci.,* **15,** 390.

SELZER, G., SHERMANN, G., CALLIHAN, T. & SWARTZ, T. (1979*b*). Primary small intestinal lymphoma and heavy chain disease. A study of 43 cases from a pathology department in Israel. *Isr. J. Med. Sci.,* **15,** 111.

TINAZTERE, B., ISIK, T. & TINAZTERE, K. (1973). Burkitt's lymphoma. *Turk. J. Pediat.,* **15,** 129.

ZIEGLER, J.L. (1977). Treatment results of 54 American patients with Burkitt's lymphoma are similar to the African experience. *N. Engl. J. Med.,* **297,** 75.